# Physicochemical characteristics of larval habitats and biodiversity of mosquitoes in one of the most important metropolises of southern Iran

**DOI:** 10.1016/j.heliyon.2023.e22754

**Published:** 2023-11-23

**Authors:** Parisa Soltan-Alinejad, Shima Bahrami, Davood Keshavarzi, Marziae Shahriari-Namadi, Amin Hosseinpour, Aboozar Soltani

**Affiliations:** a Department of Medical Entomology and Vector Control, School of Health, Shiraz University of Medical Sciences, Shiraz, Iran; b Department of Environmental Health Engineering, School of Health, Shiraz University of Medical Sciences, Shiraz, Iran; c Department of Medical Entomology and Vector Control, School of Health, Tehran University of Medical Sciences, Tehran, Iran; d Research Center for Health Sciences, Institute of Health, Department of Medical Entomology and Vector Control, School of Health, Shiraz University of Medical Sciences, Shiraz, Iran

**Keywords:** Physicochemical characteristics, Culicidae, Habitats, Biodiversity, Iran

## Abstract

The present study aimed to investigate the roles of the physicochemical characteristics of larval habitats in biodiversity and other bionomic factors of mosquitoes in Shiraz.

The physical parameters of all habitats were recorded separately. The collected mosquito larvae were identified based on morphological characters. The water samples of larval habitats were analyzed for Biochemical Oxygen Demand (BOD, mg/L), Chemical Oxygen Demand (COD, mg/L), pH, alkalinity, turbidity, total hardness (mg/L), Electrical Conductivity (EC, μS/cm), Total Dissolved Solids (TDS, mg/L), Cl2 (mg/L), and water temperature (°C). In addition, three main indices were used for surveying biodiversity.

A total of 1229 larvae were collected from April to September 2018 and May to August 2019. Seven medically important mosquito species were identified morphologically. *Culex quinquefasciatus* and *Cx. laticinctus* had the highest distribution and abundance. Ecological results showed that the richness and diversity of species were higher and more stable in natural sites than in manmade places. The optimum BOD, COD, alkalinity, TDS, EC, pH, and temperature of water for mosquitoes of the studied areas were 140 mg/L, 360 mg/L, 160 mg/L, 420 mg/L, 840 μS/cm, 8.3, and 24 °C, respectively. Most mosquitoes tended to live in manmade, temporary, and sunny larval habitats with turbid water.

The results provided a better understanding of the biology and ecology of mosquitoes as the most important group of disease vectors to humans and animals. Hence, they could be used to apply some safer and more environmentally friendly methods for mosquito control.

## 1 Introduction

Most vector-borne disease agents, such as arboviral diseases like Dengue Virus (DENV), Yellow Fever Virus (YFV), Eastern Equine Encephalitis Virus (EEEV), Chikungunya Virus (CHIKV), Zika Virus (ZIKV), West Nile Virus (WNV), and Sindbis Virus (SINV) and some parasitical diseases like filariasis and malaria, are transmitted by mosquitoes [1]. There are eight genera and 70 species of mosquitoes in Iran [2,3]. Some species prefer ovipositing in habitats with vegetation, whereas some prefer open larval habitats for oviposition [[4], [5], [6], [7]]. Aquatic habitats could be swamps, rice fields, pools, edges of streams, grassy ditches, or some artificial habitats such as containers [1,4].

The physicochemical features of larval habitats are considered key factors in laying eggs by female mosquitoes and larvae development [8]. Some of these factors include pH, temperature, ammonia, calcium, phosphate, and chloride concentration that influence the survival and distribution of mosquito species [5,9]. For example, the best condition for the development of many mosquito species has been recorded at a water pH of 3.3–10.5. Therefore, changing water features can create favorable or unfavorable conditions for mosquitoes larvae [10,11]. In addition to determining mosquito species, the density of larvae can be estimated by the physicochemical characteristics of larval habitats [8].

Shiraz, as the capital of Fars province, is located in south-central Iran and is amongst the fifth most populous cities of the country. From the historical point of view, this city is one of the important parts of the country, which attracts many tourists annually. Up to now, *Cx. pipiens, Cx. sinaiticus, Cx. bitaeniorhynchus,Cx. laticinctus, Cx. tritaeniorhynchus, Cx. torrentium, Cx. theileri, Cx. mimeticus, Cx. quinquefasciatus,* and *Cs. longiareolata* of Culicidae have been reported in Fars province [[12], [13], [14], [15]].

Given the geographical situation of Fars province, several mosquito-borne diseases have been reported from this province until now. For instance, bird malaria has been microscopically recorded in Shiraz and Fasa county, Fars province [16,17]. In addition, bovine ephemeral fever, the main vectors of which are probably *Aedes*, *Anopheles*, and *Culex* has been reported in cattle in Fras province [18]. Moreover, WNV IgG antibody was isolated from the human sera in Fars province, which showed that the people in this area were exposed to the virus [19].

Generally, having information about mosquito species and their distribution can have a great role in controlling vector-borne diseases. Determination of mosquito biodiversity also plays an important role in making good decisions for controlling them through modification and environmental manipulation alongside chemical control [20]. Thus, the present study aims to assess the effects of the physicochemical conditions of larval habitats and biodiversity and richness of medically important mosquito species in Shiraz for the first time.

## 2 Materials and methods

### 2.1 Determination of sampling sites

**Fig. 1:**
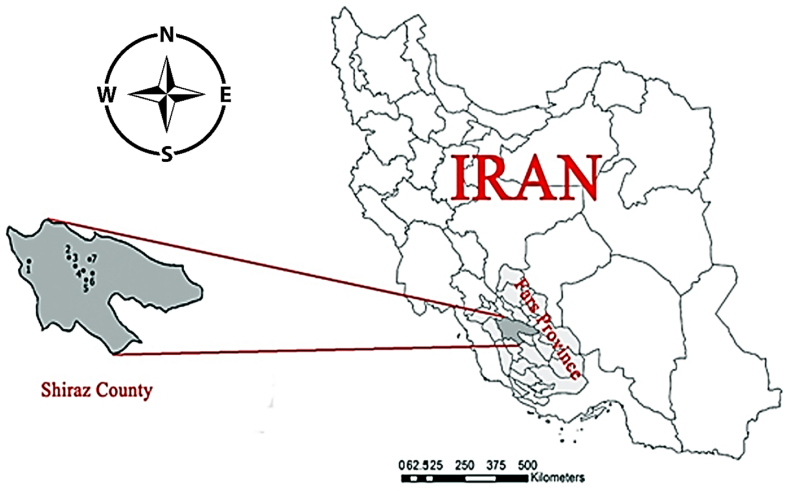
Map of sampling locations in various parts of Shiraz County, Fars Province, Southern Iran: 1) Arjan Dasht; 2) Chamran school; 3) Azadi Park; 4) Khoshk River; 5) School of Public Health; 6) Sa'di tomb; 7) Veterinary Medicine College.

Shiraz is the capital of Fars province, which is located at latitude 29.61 and longitude 52.53 and 1545 m above the sea level. The total average annual precipitation has been reported to be 305.6 mm (about 12 inches). In this study, sampling sites were selected based on various factors, including type of larval habitats, water availability, sunlight penetration, water flowing, and water transparency, for considering the probable effects of physical conditions on mosquitoes' diversity and richness. Based on these data, seven larval habitats with a variety of environmental and ecological conditions were selected for sampling in Shiraz for 10 months during rainy and dry seasons from April to September 2018 and May to August 2019. To ensure that all larvae were caught, each larval habitat was surveyed two times for 10 months. The selected sites with their geographical coordinates were as follows: Azadi Park (29°37′46.4"N 52°32′19.9"E), Khoshk River 29°37′07.9"N 52°33′18.1"E), Sa'di tomb (29°37′21.6"N 52°34′59.5"E), Veterinary Medicine College (29°36′49.0"N 52°33′28.8"E), Chamran School (29°39′54.5"N 52°29′20.1"E), Dasht Arjan (29°41′33.4"N 52°03′05.6"E), and School of Public Health affiliated to Shiraz University of Medical Sciences (29°35′32.1"N 52°33′37.9"E). During the study, the coordinates of each larval habitat were recorded using the Global Positioning System (GPS). All these sites have been shown in Fig. 1.

Physical parameters, such as type of larval habitats (natural or manmade), water availability (temporary or permanent), sunlight penetration (shady, partially sun-lighted, or sun-lighted), water flowing, and water transparency (clear, slightly turbid, or turbid), were recorded separately for each larval habitat. The physical parameters of the sampling larval habitats have been presented in Table 1, Table 2, Table 3, Table 4.

### 2.2 Mosquito collection and water sampling

#### 2.2.1 Mosquito larval sampling

Mosquito larvae are heterogeneously distributed in larval habitats. This problem has been considered by Service (1993). In the present study, the mosquito larvae were collected using a standard dipping method (20 dips of 350 mL dipper each, 13 cm diameter per unit) (BioQuip®, Rancho Dominguez, CA) [21] in each larval habitat. In the small areas where the dipping method could not be performed, a 1 mL transfer pipette was used to pick up mosquito larvae and pupae. Mosquito larvae were collected from fixed larval habitats, such as river edges, water channels, and artificial pools, in the seven selected places.

#### 2.2.2 Water sampling

In order to do the physicochemical analysis, the water samples of each larval habitat were collected in 100 mL plastic bottles. All the bottles were labeled according to the sampling site and date. The temperature was also recorded for all places. The samples were kept in a cold box to be transferred to the laboratory of Environmental Health Engineering in School of Health for analysis. The results have been presented in Table 5. It should be noted that the means and standard deviations (M±SD) of all physicochemical characteristics were calculated for each mosquito species based on the studied area (Table 6).

### 2.3 Mosquito identification

All the samples were transferred to the insectarium of the Medical Entomology laboratory in Shiraz University of Medical Sciences to be kept for the development of the first and second instar larvae and emergence of pupae (12:12 light/dark cycle, 25–27 °C, and 70 % humidity). The third- and fourth-instar larvae were stored in lacto-phenol and mounted on glass slides using Berlese's fluid (Gum chloral) in the laboratory. All collected mosquitoes were identified according to valid taxonomic keys [22,23] based on morphological characteristics. The number of collected mosquito species from each habitat was recorded, as well.

### 2.4 Physicochemical characteristics of the larval habitats' water

The physicochemical parameters, including Biochemical Oxygen Demand (BOD, mg/L), Chemical Oxygen Demand (COD, mg/L), Potential of Hydrogen (pH), alkalinity, turbidity [Nephelometric Turbidity Unit (NTU)], total hardness (mg/L), Electrical Conductivity (EC, μS/cm), Total Dissolved Solids (TDS, mg/L), Cl_2_ (mg/L), and water temperature (°C), were measured according to standard procedures [24]. All physicochemical characteristics were measured in the laboratory, except for water temperature and ${\text{Cl}}_{2}$. Total hardness, TDS, pH, and EC were measured using titration. Finally, chlorine levels were measured by DPD tablets (N, N-diethyl-*p*-phenylenediamine) (Lovibond®). All experiments were repeated twice for increasing the accuracy.

The multiparameter pH meter (Metrohm 827) was checked for calibration before measurement. To measure turbidity, the HACH 2100Q turbidimeter was used. In addition, the HACH DR5000 spectrophotometer and HACH DRB200 COD reactor were used for assessment of COD. Besides, BOD was evaluated using the WTW respirometric BOD measuring system (OxiTop®). Finally, a conductivity meter (WTW Cond 720, Germany) was used for measuring EC.

### 2.5 Diversity of species

The species diversity in the two groups was assessed using the three measures of Hill numbers (or “the effective number of species”) of order q; i.e., species richness (q = 0), Shannon diversity (q = 1), and inverse Simpson diversity (q = 2). It was hypothesized that species richness and diversity metrics would show differences between the groups. Hill numbers, as a static method, quantify a community's species diversity [25]. The sample size-based rarefaction and extrapolation (R/E) curves with 95 % confidence intervals based on a bootstrap method were used to compare the groups with respect to the species diversity. The analysis was performed with an R package (iNEXT) provided by Chao et al. (2014). The detailed formulas and information for the Hill numbers and iNEXT package can be found elsewhere [25].

### 2.6 Statistical analysis

The means and standard deviations of the physicochemical characteristics were calculated by the Statistical Package for the Social Sciences (SPSS) software (version 22 for windows, SPSS Inc.). The physicochemical features of the larval habitats' water were compared using Kruskal-Wallis test. In addition, Pearson's correlation coefficient was used to determine the relationships between mosquitoes and physicochemical factors.

## 3 Results

### 3.1 Distribution of mosquito species sampling sites

In this study, seven larval habitats were surveyed for mosquito species sampling. The sample contained 1229 larvae, including seven species of two genera of Culicinae subfamilies. The identified species were *Culex. laticinctus, Cx. perexiguus, Cx. tritaeniorhynchus, Cx. pipiens, Cx. antennatus, Cx. quinquefasciatus,* and *Culiseta longiareolata*, and their frequencies have been shown in Table 1. Accordingly, the highest (36.9 %) and lowest (1.5 %) number of specimens were related to Khoshk River and Azadi Park, respectively. Additionally, *Cx. quinquefasciatus* (44.3 %) and *Cx. laticinctus* (23.4 %) were dominant and pre-dominant species in the selected sites, respectively. The lowest abundance of the collected mosquito species belonged to *Cx. tritaeniorhynchus* (3.3 %) and *Cx. antennatus* (1.9 %) (Table 2). The results indicated that *Cx. quinquefasciatus* was collected from five sampling sites. In other words, these species had the highest distribution in Shiraz. The lowest distribution belonged to *Cx. tritaeniorhynchus* that was recorded only from one habitat; i.e., Khoshk River. All seven reported species in the current study, including *Cx. laticinctus, Cx. perexiguus, Cx. tritaeniorhynchus, Cx. pipiens, Cx. antennatus,* and *Cs. longiareolata*, were recorded in Shiraz for the first time. Nevertheless, these species were previously reported from different counties around Fars province.

**Table 1:** The locality and number of captured Culicidae larvae in the selected sites, Shiraz, Fars province, 2018–2019.

Species	Sampling locations							
	Azadi Park	Khoshk River	Sa'di tomb	Veterinary Medicine College	Chamran School	Dasht Arjan	School of Public Health	Total larvae
***Cx. Laticinctus***	15	179	82	–	–	12	–	**288**
**Cx. perexiguus**	–	28	–	–	26	–	74	**128**
***Cx. tritaeniorhynchus***	–	40	–	–	–	–	–	**40**
**Cx. pipiens**	–	70	50	–	21	10	–	**151**
***Cx. antennatus***	–	–	–	–	–	5	18	**23**
***Cx. quinquefasciatus***	4	136	68	–	–	10	326	**544**
***Cs. longiareolata***	–	–	–	20	–	35	–	**55**
Total	**19**	**453**	**200**	**20**	**47**	**72**	**418**	**1229**

**Table 2:** The percentage of Culicidae species in Shiraz, Fars province, 2018–2019.

Species	Total number	Percentage
***Cx. laticinctus***	288	23.4
**Cx. perexiguus**	128	10.4
***Cx. tritaeniorhynchus***	40	3.3
**Cx. pipiens**	151	12.3
***Cx. antennatus***	23	1.87
***Cx. quinquefasciatus***	544	44.26
***Cs. longiareolata***	55	4.47
Total	**1229**	**100**

### 3.2 Diversity of mosquito species

**Fig. 2:**
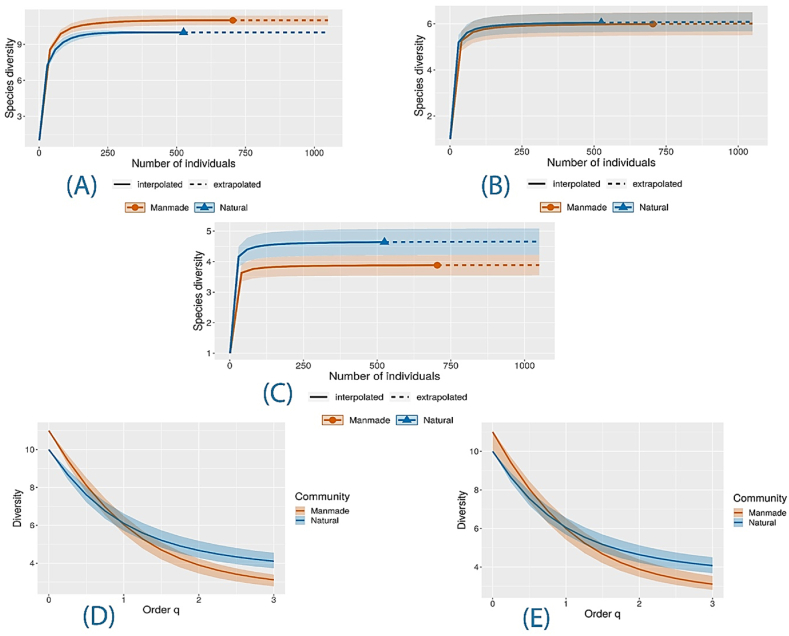
(A) Comparison of sample-size-based rarefaction (solid lines) and extrapolation (dashed lines) of mosquito species diversity for Hill numbers (q = 0) in two sites; (B) Comparison of sample-size-based rarefaction (solid lines) and extrapolation (dashed lines) of mosquito species diversity for Hill numbers (q = 1) in two sites; (C) Comparison of sample-size-based rarefaction (solid lines) and extrapolation (dashed lines) of mosquito species diversity for Hill numbers (q = 2) in two sites; (D) Diversity profile estimated for Hill numbers (q = 0, 1, 2) in two sampling sites; (E) Diversity profile emprical for Hill numbers (q = 0, 1, 2) in two sampling sites.

The results of biodiversity analysis demonstrated that the richness and diversity of the species were higher and more stable in natural habitats than in manmade places. The graphs of q0-Species richness, q1-Shannon diversity, and q2-Simpson diversity have been presented in Fig. 2(A–E).

### 3.3 Physical characteristics of the larval habitats

All species reported from the habitats had common physical characteristics; i.e., natural larval habitats and sun-lighted sites with turbid, stagnant, and temporary water (Table 3).

**Table 3:** The physical characteristics of the sampling larval habitats, Shiraz, Fars province, 2018–2019.

Physical characteristics	Sampling locations						
	Azadi Park	Khoshk River	Sa'di tomb	Veterinary Medicine College	Chamran School	Dasht Arjan	School of Public Health
Type of breeding site	Manmade	Natural	Manmade	Manmade	Manmade	Natural	Manmade
Water availability	Temporary	Permanent	Temporary	Temporary	Temporary	Permanent	Temporary
Sunlight penetration	Shady	Sun-lighted	Partially sun-lighted	Shady	Shady	Sun-lighted	Shady
Water flowing	Stagnant	Stagnant	Stagnant	Stagnant	Stagnant	Stagnant	Stagnant
Water transparency	Clear	Turbid	Slightly turbid	Slightly turbid	Clear	Turbid	Clear

**Table 4:** The percentage of species composition by different physical parameters in each habitat, Shiraz, Fars province, 2018–2019.

Physical parameters	Habitat characteristics	Kind of species							
		*Cx. laticinctus* (%)	Cx. perexiguus (%)	*Cx. tritaeniorhynchus* (%)	Cx. pipiens (%)	*Cx. antennatus* (%)	*cx. quinquefasciatus* (%)	*Cs. longiareolata* (%)	Total (%)
**Type of breeding site**	Natural	66.3	21.9	100	53.0	21.7	26.8	63.6	**42.7**
	Manmade	33.7	78.1	–	47.0	78.3	73.2	36.4	**57.3**
**Water availability**	Temporary	33.7	78.1	–	47.0	78.3	73.2	36.4	**57.3**
	Permanent	66.3	21.9	100	53.0	21.7	26.8	63.6	**42.7**
**Sunlight penetration**	Shady	5.2	78.1	–	13.9	78.3	60.7	36.4	**41.0**
	Partially sun-lighted	28.5	–	–	33.1	–	12.5	–	**16.3**
	Sun-lighted	66.3	21.9	100	53.0	21.7	26.8	63.6	**42.7**
**Water flowing**	Stagnant	100	100	100	100	100	100	100	**100**
	Flow	–	–	–	–	–	–	–	
**Water transparency**	Clear	5.2	78.1	–	13.9	78.0	60.7	–	**39.4**
	Slightly turbid	28.5	–	–	33.1	–	12.5	36.4	**17.9**
	Turbid	66.3	21.9	100	53.0	21.7	26.8	63.6	**42.7**

**Table 5:** The physicochemical characteristics of larval habitats, Shiraz, Fars province, 2018–2019.

Physicochemical parameters	Sampling locations						
	Azadi Park	Khoshk River	Sa'di tomb	Veterinary Medicine College	Chamran School	Dasht Arjan	School of Public Health
**Temperature (°C)**	23	24	23	20	22	26	25
**Potential of hydrogen (**pH**)**	8.2	8.3	8.3	8.2	8.5	8.3	7.5
**Total dissolved solids (TDS) (mg/L)**	320	420	520	540	1130	600	462
**Electrical conductivity (EC) (μS/cm)**	640	840	1030	1080	2150	1200	960
**Alkalinity (mg/L)**	134	160	140	144	232	164	120
**Total hardness (mg/L)**	240	134	520	280	700	340	378
**Turbidity (NTU)**	2.19	7.2	3.05	3.43	4.85	3.5	2.83
**Chemical oxygen demand (COD) (mg/L)**	65	360	91	198	195	415	84
**Biochemical oxygen demand (BOD) (mg/L)**	29	140	56	98	96	158	35

**Table 6:** The mean and standard division (M±SD) of the physicochemical parameters related to the larval habitats of the collected species, Shiraz, Fars province, 2018–2019.

Physicochemical parameters	Kind of species						
	Cx. laticinctus	Cx. perexiguus	Cx. tritaeniorhynchus	Cx. pipiens	Cx. antennatus	Cx. quinquefasciatus	Cs. longiareolata
Temperature (°C)	24 ± 1.4 23–26	23.66 ± 1.52 22–25	24	23.75 ± 1.70 22–26	25.5 ± 0.7 25–26	24.2 ± 1.3 23–26	23 ± 4.2 20–26
pH	8.2 ± 0.05 8.2–8.3+	8.1 ± 0.52 7.5–8.5	8.3	8.35 ± 0.1 8.3–8.5	7.9 ± 0.5 7.5–8.3	8.12 ± 0.34 7.5–8.3	8.25 ± 0.07 8.2–8.3
TDS	465 ± 121.5 320–600	670.66 ± 398.34 420–1130	420	667.5 ± 317 420–1130	531 ± 97.5 462–600	428.4 ± 146.8 320–600	570 ± 42.42 540–600
EC	927 ± 241.5 640–1200	1316.66 ± 724.17 840–2150	840	1305 ± 582.2 840–2150	1080 ± 169.7 960–1200	934 ± 209.71 640–1200	1140 ± 84.85 1080–1200
Alkalinity	154.5 ± 13.79 134–164	170.66 ± 56.75 120–232	160	174 ± 40 140–232	142 ± 31.1 120–164	143.6 ± 18.35 120–164	154 ± 14.14 144–164
Total hardness	308.5 ± 164.1 134–520	404 ± 283.89 134–700	134	423.5 ± 242.5 134–700	359 ± 26.87 340–378	322.4 ± 145.54 134–520	310 ± 42.42 280–340
Turbidity	3.98 ± 2.21 2.19–7.2	4.96 ± 2.18 2.83–7.2	7.2	4.65 ± 1.86 3.05–7.2	3.1 ± 0.47 2.83–3.5	3.75 ± 1.98 2.19–3.5	3.46 ± 0.04 3.43–3.5
COD	232.75 ± 180.40 65–415	465 ± 335.07 194–840	360	265.25 ± 149.1 91–415	249.5 ± 234 84–415	203 ± 169.8 65–415	306.5 ± 153.44 198–415
BOD	70.75 ± 48.02 29–140	90.33 ± 52.72 35–140	140	87.5 ± 39.5 56–140	46.5 ± 16.26 35–58	64.2 ± 44.09 29–58	78 ± 28.28 58–98

**Table 7:** The associations between the collected mosquitos and other species in Shiraz, Fars province, 2018–2019.

		Cx. laticinctus	Cx. perexiguus	Cx. tritaeniorhynchus	Cx. pipiens	Cx. antennatus	Cx. quinquefasciatus	Cs. longiareolata
*Cx. laticinctus*	Pearson correlation	1	.671	.929^b^	.975^b^	.480	.725^a^	.524
	Sig. (2-tailed)		.068	.001	.000	.229	.042	.182
							
*Cx. perexiguus*	Pearson correlation	.671	1	.611	.711^a^	.920^b^	.968^b^	.511
	Sig. (2-tailed)	.068		.108	.048	.001	.000	.196
							
*Cx. tritaeniorhynchus*	Pearson correlation	.929^b^	.611	1	.854^b^	.379	.632	.401
	Sig. (2-tailed)	.001	.108		.007	.354	.092	.325
							
*Cx. pipiens*	Pearson correlation	.975^b^	.711^a^	.854^b^	1	.519	.738^a^	.578
	Sig. (2-tailed)	.000	.048	.007		.188	.036	.133
							
*Cx. antennatus*	Pearson correlation	.480	.920^b^	.379	.519	1	.933^b^	.582
	Sig. (2-tailed)	.229	.001	.354	.188		.001	.130
							
*Cx. quinquefasciatus*	Pearson correlation	.725^a^	.968^b^	.632	.738^a^	.933^b^	1	.519
	Sig. (2-tailed)	.042	.000	.092	.036	.001		.188
							
*Cs. longiareolata*	Pearson correlation	.524	.511	.401	.578	.582	.519	1
	Sig. (2-tailed)	.182	.196	.325	.133	.130	.188	
							

a Correlation is significant at the 0.05 level (2-tailed).

b Correlation is significant at the 0.01 level (2-tailed).

The frequency of larvae was 42.7 % in natural sites and 57.3 % in manmade places. Therefore, the frequency of species was a little higher in temporary oviposition sites. Moreover, 525 (42.7 %), 504 (41 %), and 200 (16.3 %) larvae were collected from sun-lighted, shady, and partially sun-lighted sites, respectively. Additionally, most larvae were collected from turbid (42.7 %) and clear (39.4 %) water. Other data have been presented in details in Table 4.

The results indicated a significant difference among the habitats with different physical features regarding the number and species of the collected mosquitoes (p = 0.032). Based on the results, *Cx. tritaeniorhynchus* (100 %), *Cx. laticinctus* (66.3 %), and *Cs. longiareolata* (63.6 %) were the most frequent species in natural sites with permanent water. The results also demonstrated that all species were dispersed in natural and manmade sites, except for *Cx. tritaeniorhynchus* (100 %) that was recorded only from natural oviposition sites. Furthermore, all seven identified species (47.2 %) were collected from sun-lighted sites and turbid water. Therefore, these two sites had the highest species diversity.

### 3.4 Physicochemical analysis

The results of the physicochemical characteristics of all collected water samples from different habitats have been presented in Table 5. Besides, the calculated physicochemical properties for each mosquito species based on the studied area have been shown in Table 6.

Totally, 36.9 % of the larvae were collected from the water with BOD = 140 mg/L, COD = 360 mg/L, and NTU = 2.19, as the most preferred conditions for mosquitoes in this area. Conversely, 1.5 % of the larvae belonged to the water with BOD = 29 mg/L, COD = 65 mg/L, and NTU = 7.2, as the least desired habitat. From the collected mosquitoes, 36.9 % and 1.5 % belonged to the habitats with 134 mg/L and 240 mg/L total water hardness, respectively. In addition, 36.9 % and 34 % of the larvae belonged to the water alkalinity of 160 and 120 mg/L. Furthermore, 1.5 %, 1.6 %, and 3.8 % of the mosquitoes were collected from the water alkalinity of 16, 20, and 47 mg/L, respectively. Moreover, about 37 % and 34 % of the larvae belonged to the water with EC of 840 and 960 μS/cm, respectively. The lowest percentage of larvae (1.5 %, 1.6 %, and 3.8 %) were reported from oviposition sites with EC of 640, 1080, and 2150 μS/cm, respectively. The frequency of larvae was 59 % in the water with pH = 8.3, but 3.2 % in the water with pH = 8.2. Finally, the frequency of species was 36.9 % and 1.5 % in the water with TDS of 420 and 320 mg/L, respectively.

The findings indicated a significant difference among the habitats with different physicochemical parameters concerning the collected mosquito species (p = 0.01). The optimum BOD of water, which had the highest abundance of species, was calculated to be 140 mg/L. Under this condition, the collected species were *Cx. tritaeniorhynchus* (100 %), *Cx. laticinctus* (62.2 %), *Cx. pipiens* (46.4 %), *Cx. quinquefasciatus* (25 %), and *Cx. perexiguus* (21.9 %), while *Cs. longiareolata* and *Cx. antennatus* were not recorded. In addition, all seven species were detected in COD = 360 mg/L, except for *Cs. longiareolata* and *Cx. antennatus*. In this regard, oviposition sites with COD = 65 mg/L were least preferred for mosquito larvae (species abundance = 1.5 %). Based on the results, Khoshk River had the highest water turbidity (7.2 NTU). The results also revealed that 36.9 % of all collected species tended to live in turbid water (100 % of *Cx. tritaeniorhynchus*, 62.2 % of *Cx. laticinctus*, 46.4 % of *Cx. pipiens*, 25 % of *Cx. quinquefasciatus*, and 21.9 % of *Cx. Perexiguus*).

The study findings showed an inverse relationship between the species diversity and water hardness. Accordingly, *Cx. tritaeniorhynchus* was often found at the lowest total water hardness (134 mg/L) in Khoshk River, but *Cs. longiareolata* and *Cx. antennatus* were not reported from the places with this degree of hardness. Only *Cx. pipiens* (13.9 %) and *Cx. perexiguus* (20.3 %) were collected from oviposition sites with the highest total hardness of water (700 mg/L) in Chamran School. Furthermore, the optimum alkalinity for all species was 120–160 mg/L.

Almost all species were collected from aquatic sites with EC = 840 μS/cm, except for *Cs. longiareolata*. In fact, *Cs. longiareolata* females tend to lay eggs in water with EC = 1080 and 1200 μS/cm. The highest EC of water (2150 μS/cm) was reported from Chamran School. Only two species, including *Cx. pipiens* (13.9 %) and *Cx. perexiguus* (20.3 %), were collected from the water with this level of EC. Moreover, the obtained optimum pH for mosquitoes to lay eggs was 8.3, and all seven identified species were caught in oviposition sites with this condition.

*Cx. pipiens* (13.9 %) and *Cx. perexiguus* (20.3 %) were found in the water with the highest TDS (11300) in Chamran School. Five species of the collected larvae, including *Cx. tritaeniorhynchus* (100 %), *Cx. laticinctus* (62.3 %), *Cx. pipiens* (46.4 %), *Cx. quinquefasciatus* (25 %), and *Cx. perexiguus* (21.9 %), were recorded in TDS = 420 mg/L. *Cs. longiareolata* and *Cx. antennatus* would rather live in the water with a higher level of TDS. The larvae frequency decreased from 453 (36.9 %) in TDS = 420 mg/L to 20 (1.6 %) in TDS = 540 mg/L. Only some specific species with a different frequency were recorded at TDS = 600 mg/L, including *Cx. laticinctus* (4.2 %), *Cx. pipiens* (6.6 %), *Cx. antennatus* (21.7 *%), Cx. quinquefasciatus* (1.8 %), and *Cs. longiareolata* (63.6 %). Furthermore, 13.9 % of *Cx. pipiens* and 20.3 % of *Cx. prexiguus* were reported from the larval habitats with TDS = 1130 mg/L.

*Cs. longiareolata* (36.4 %) was the only species that was found at the lowest temperature (20 **°C**) in the current study. The optimum temperature for five species, including *Cx. tritaeniorhynchus* (100 %)*, Cx. laticinctus* (62.3 %), *Cx. perexiguus* (21.9 %), *Cx. pipiens* (46.4 %), and *Cx. quinquefasciatus* (25 %), was 24 °C. However, *Cs. longiareolata* and *Cx. antennatus* were not recorded at this temperature.

### 3.5 The correlations between mosquito species

The results of the statistical analysis of the relationships between different species of mosquitoes collected from the selected oviposition sites have been presented in details in Table 7. Accordingly, *Cx. laticinctus* had a significant, strong correlation with *Cx. tritaeniorhynchus* (r = 0.929, p = 0.001) and *Cx. pipiens* (r = 0.975, p = 0.001) and a significant correlation with *Cx. quinquefasciatus* (r = 0.725, p = 0.042). In addition, a significant correlation was observed between *Cx. perexiguus* and three other species, including *Cx. pipiens, Cx. quinquefasciatus,* and *Cx. antennatus* (p < 0.05). *Cx. tritaeniorhynchus* also showed a significant, strong correlation with *Cx. laticinctus* and *Cx. pipiens* (p < 0.01). However, there was no significant correlation between *Cs. longiareolata* and other species. The results revealed a significant correlation between *Cx. pipiens* and four species, including *Cx. laticinctus, Cx. perexiguus, Cx. tritaeniorhynchus,* and *Cx. quinquefasciatus* (p < 0.05). Besides, *Cx. antennatus* showed a significant correlation with two species; i.e., *Cx. perexiguus* and *Cx. quinquefasciatus* (p < 0.01). However, *Cx. quinquefasciatus* had a significant correlation with four other species (p < 0.05). *Cs. longiareolata* did not show any significant correlations with any of the other collected mosquitoes (p > 0.05).

## 4 Discussion

In this investigation, a total of 1229 Culicidae larvae, including seven species and two genera, were collected from different selective larval habitats in Shiraz. In 2017, Keshavarzi et al. investigated the monthly prevalence and diversity of Culicidae in Fars province. Their studied areas included Khonj, Larestan, Mohr, Lamerd, Darab, and Zarindasht counties [15]. However, they did not cover Shiraz as the capital of Fars province. In another research, Soltani et al. (2017) reported the fauna and active seasons of mosquitoes in the west of Fars province. They collected mosquitoes from Banab, Dadenjan, Mahkoya, Dehrod, and Zanjiran, as five counties in the west of Fars province [13]. In 2019, Hoseini et al. collected Culicidae for performing a molecular-based survey on *Rickettsia* spp. and *Coxiella burnetii* from Qir and Karzin counties, Fars province [14]. Considering the fact that other researchers have not covered Shiraz so far, the present study is of particular importance because it examined the impact of various environmental and ecological factors on the bioecology and biodiversity of mosquitoes for the first time.

The results demonstrated that in the studied area, mosquitoes preferred manmade and sun-lighted aquatic habitats with temporary and turbid water for growth and reproduction. Temperature also played a key role in the development of the Culicidae larvae. Thus, having information about the temperature of oviposition sites could be helpful for designing appropriate control methods [26]. In some geographical areas, especially in temperate regions, temperature fluctuates widely during the day and night that can affect the survival and reproduction of mosquitoes [27]. Therefore, this parameter varies widely in such investigations.

The current study findings revealed a negative correlation between the TDS of water and the frequency of species. In another study, a similar relationship was reported between the TDS of water and the frequency of *Cx. quinquefasciatus* larvae in Puerto Rico [28]. Finding the correlation between the physicochemical characteristics of larval habitats and mosquito species is essential in order to organize mosquito control measures more effectively.

### 4.1 Culex tritaeniorhynchus

Among all collected mosquitoes in this study, *Cx. tritaeniorhynchus* (3.3 %) had the lowest frequency. In a similar study, Hanafi-bojd et al. (2017) collected *Cx. tritaeniorhynchus* (95.3 %) from natural oviposition sites in Bashagard county, Hormozgan province [20]. Azari-Hamidian et al. (2007) also reported that 80.59 % of this species were collected from natural larval habitats in Guilan province [29]. In another study conducted in 13 provinces of Iran in 1987, Zaim et al. reported 24 % of *Cx. tritaeniorhynchus* from natural habitats [30]. Many studies have indicated this species as the dominant mosquito in paddy fields in the world [31,32]. One of the oviposition sites in the present study; i.e., Khoshk River, as a natural place looked turbid and muddy. Muddy rivers have been considered the best place for *Cx. tritaeniorhynchus* to lay eggs, grow, and reproduce [33]. Thus, Khoshk River with the highest water turbidity (NTU = 7.2) could be a suitable larval habitat for this species compared to other sites. However, Moradi-Asl et al. (2018) reported *Cx. tritaeniorhynchus* from natural sites (87.5 %) with permanent (71 %), partially sun-lighted (45 %), and stagnant water (84.5 %) in Ardabil province, northwest of Iran [34]. This difference could be attributed to such factors as geographical and climatic differences between the two sampling places.

One of the important chemical compounds of larval habitats that plays a critical role in total water hardness, insects growth, and female mosquito attraction is calcium carbonate (CaCO3) [35]. In line with the present study, Hanafi-Bojd et al. performed a research in 2017 and showed a total water hardness of 231.3 ± 103 mg/L for *Cx. tritaeniorhynchus*, which was the lowest amount of all their selected larval habitats in Bashagard county, Hormozgan province [20]. As a result, it can be concluded that this species tends to lay eggs and grow in the water with a low total hardness.

In the present study, two physicochemical parameters; i.e., BOD and COD, were checked for *Cx. tritaeniorhynchus* for the first time in Iran. Similarly, Mukhtar et al. (2006) reported *Cx. tritaeniorhynchus* as the most predominant species in wastewater sites in Pakistan [36]. They stated that this species mostly chose nutrition-rich sites to lay eggs, because wastewater sites could provide food for the immature stage of mosquitoes.

*Cx. tritaeniorhynchus* is an important vector for the transmission of the Japanese Encephalitis Virus (JEV) [37] and Rift Valley Fever Virus (RVFV) [38]. Wading birds and pigs are known as amplifying hosts for JEV [39]. Thus, the presence of these hosts, especially infected migratory seabirds, along with *Cx. tritaeniorhynchus* around wastewater sites like Khoshk River can be a serious threat for residents along the river. Infected livestock with RVFV is yet another main host for *Cx. tritaeniorhynchus*, which can have an important role in transmission of the disease to humans [40]. Because of detection of this virus in cattle and sheep from Kurdistan province in Iran [41], this arboviral disease should be taken into consideration in this area.

### 4.2 Culex quinquefasciatus

Most of the collected larva of this species (59.9 %) were found from manmade larval habitats in the School of Public Health (73.2 %). In another study performed in Bashagard county, Hormozgan province, 22.9 % of all collected larvae belonged to *Cx. quinquefasciatus*. In that study, 87.6 % of this species were collected from natural sites [20]. In another similar study carried out by Zaim et al. (1987) in Iran, 59.1 % of this species was recorded from natural sites [30]. Hanafi-Bojd et al. (2017) maintained that these mosquito larvae were from fully sun-lighted sites (63.3 %) with temporary (61.0 %) and clear water (50.8 %) [20]. Zaim et al. (1987) also found the largest number of these larvae in temporary water (67.1 %), fully sun-lighted sites (70.5 %), and clear water (68.1 %) [30]. In contrast, this species was mostly collected from shady habitats in Shiraz. Based on the results, 73.2 % of this species lived in manmade sites that were filled with well water and 59.9 % tended to live in a total hardness of 378 mg/L and pH of 7.5, which was the lowest pH among the selected sites. In a previous study conducted in Hormozgan province, *Cx. quinquefasciatus* was identified at a pH of 6.9 that was the lowest one, while the total hardness was at the highest level (353.8 mg/L) [20]. These results indicated the probability of a negative association between the water pH and the abundance of mosquito larvae in aquatic habitats. In contrast to the present investigation, *Cx. quinquefasciatus* has been frequently reported from sewage-contaminated water bodies. Therefore, the high frequency of this species in clean water might be due to the entry of some chemicals, including pesticides, into wastewater and their detrimental effects on mosquitoes, which requires further investigation. *Cx. quinquefasciatus* has been identified as a potential vector for transmission of lymphatic filariasis and WNV [42]. Given that WNV has been reported in Iran previously [43], these manmade sites can be considered potential and ideal oviposition sites for this vector. Hence, open wells should be covered in order to prevent gravid female mosquitoes from accessing these places. It should be noted that physicochemical parameters, such as BOD and COD, were measured for this species for the first time in Iran.

### 4.3 Culex laticinctus

*Cx. laticinctus* was the predominant species in Shiraz. In a study carried out by Omrani et al. (2020) in Chaharmahal and Bakhtiari province, 100 % of *Cx. laticinctus* were from natural places. They were collected from permanent water (66.7 %), muddy water (66.7 %), and sun-lighted sites (66.7 %) [27]. This species was also reported from mid- and high-altitude natural sites in Koohrang region [27]. The results of the current study were consistent with those of the previous studies.

In the present study, the abundance of larvae was negatively associated with the amount of TDS. According to BOD and COD analyses, *Cx. laticinctus* females tend to lay eggs in muddy and sewage-contaminated sites. The high level of BOD in this oviposition site can be attributed to the high level of organic matter, making it a suitable site for laying eggs and developing immature stages. Thus, such water can be a good choice for gravid female mosquitos, such as *Cx. laticinctus*. All physicochemical assessments carried out for *Cx. laticinctus* in the current study, except for pH and temperature of water, were recorded for the first time in Iran.

### 4.4 Culiseta longiareolata

Hanafi-Bojd et al. (2017) indicated that the frequency of *Cs. longiareolata* was 37.5 %, which was collected from natural sites (66.9 %) and temporary (63.9 %), fully sun-lighted (55.9 %), and turbid water (57.6 %) in Hormozgan province [20]. In 2005, Azari-Hamidian et al. recorded this species from temporary water (96.45 %) and fully sun-lighted (38.32 %) and shaded sites (35.03 %). Additionally, 100 % of the specimens were sampled from natural sites [44]. Abai et al. (2016) also collected all specimens (32.12 %) from natural sites [45]. Moreover, Sofizadeh et al. (2018) identified all specimens from temporary water, sun-lighted sites, and clear water [46]. Zaim et al. (1987) also reported *Cs. longiareolata* from natural sites (78 %), permanent water (61.1 %), fully sun-lighted sites (71.9 %), and clear water (89.3 %) [30]. In 2020, Omrani et al. conducted an investigation in Chaharmahal and Bakhtiari province and collected this species from natural sites (58.6 %), temporary and clear water (89.7 %), and sun-lighted sites (72.4 %) [27]. Similarly, Hanafi-Bojd et al. (2017) reported that most of them were from turbid water. In contrast, Sofizadeh et al. (2018), Omrani et al. (2020), and Zaim et al. (1987) collected this species mostly from clear water. These differences might be attributed to the high adaptability of this species in diverse types of aquatic habitats in different geographical conditions of the country.

In 2017, Hanafi-Bojd et al*.* conducted a research in Hormozgan province and found the following features: TDS = 1003.4 ± 308.4 mg/L, EC = 965.8 ± 24.6 μS/cm, alkalinity = 146.2 ± 4.1 mg/L, total hardness = 270.4 ± 117.8 mg/L, NTU = 14.3 ± 6.9, and temperature = 22.3 ± 3.2 °C [20]. Abai et al. (2016) also performed a study in Qom province and reported the physicochemical characteristics as follows: temperature = 19.4 ± 1.6 °C, NTU = 0.4 ± 0.4, EC = 1429.6 ± 906.8 μS/cm, TDS = 713.6 ± 451.1 mg/L, alkalinity = 416.6 ± 120.1 mg/L, and total hardness = 469.6 ± 311.4 mg/L [45]. These features were reported by Nikookar et al. (2017) as follows: temperature = 16.05 ± 2.06 °C, NTU = 35.95 ± 73.21, EC = 761.55 ± 824.98 μS/cm, alkalinity = 231.25 ± 128.02 mg/L, and total hardness = 474.40 ± 514.07 mg/L [8]. In the present study, this species was only found at the lowest temperature (20 °C) among all the checked oviposition sites, while Omrani et al. (2020) recorded the minimum temperature (12 °C) for this species larvae in Chaharmahal and Bakhtiari province [27]. Hence, it can be concluded that this species may prefer low water temperatures and may have the ability to withstand lower temperatures in comparison to other mosquitoes.

Similar to the present study results, Abai et al. (2016) mentioned that the level of water alkalinity was high in oviposition sites. CaCO3 probably plays an important role in the physiology and life cycle of this species.

In the current research, 63.3 % of *Cs. longiareolata* was found in the water with BOD of 158 mg/L, which was the highest level of BOD among all the checked sites. This implies that *Culiseta* spp. females select their oviposition sites based on food availability [47]. On the other hand, *Cx. laticinctus* and *Cs. longiareolata* would rather lay eggs in the water with few predators, because they can detect the released kairomones from predators [48]. It should be noted that the existence of different predators in water undoubtedly depends on the physicochemical characteristics of larval habitats. In the current study, COD and BOD were measured in the oviposition sites of *Cs. longiareolata* for the first time in Iran. Given that *Cs. longiareolata* is known as one of the potential vectors for transmission of WNV, avian influenza, and brucellosis [49], especially in the suburbs, special attention should be paid to the reduction of the vector population.

### 4.5 Culex pipiens

Nikookar et al. (2017) revealed that 56.22 % of *Cx. pipiens* was seen in artificial pools in northern parts of Iran [8]. Azari-Hamidian et al. (2007) recorded the physical parameters as follows: natural sites (67.30 %), clear water (97.17 %), shaded sites (51.19 %), and temporary water (80.9 %) [29]. In addition, Ladonni et al. (2015) found 86.7 % of *Cx. pipiens* larvae in manmade sites [50]. Omrani et al. (2020) also pointed out the characteristics of *Cx. pipiens* habitats in the studied areas, including natural sites (50 %), temporary water (75 %), sun-lighted sites (50 %), and clear water (75 %) [27]. The results showed that this species had higher adaptation in a variety of sites with different physical conditions, such as water transparency, site type, and sunlight penetration.

Similar to the current study, Nikookar et al. performed a research in Mazandaran Province in 2017 and reported the physicochemical features as follows: NTU = 34.97 ± 65.15, EC = 850.10 ± 994.24 μS/cm, pH = 7.13 ± 0.42, and alkalinity = 239.63 ± 149.66 mg/L [8]. Omrani et al. (2020) also reported a pH of 7.6 ± 0.8 in Chaharmahal and Bakhtiari province [27]. Furthermore, Abai et al. (2016) conducted a study in Qom province and reported the physicochemical characteristics for *Cx. pipiens* as follows: pH = 7.1, NTU = 6.0, EC = 4240 μS/cm, TDS = 2120 mg/L, alkalinity = 410 mg/L, and total hardness = 1294.7 mg/L [45]. The amount of pH in the oviposition sites of *Cx. pipiens* was higher in the present study in comparison to the previous studies performed in different parts of the country. This phenomenon might be related to the geographical and climatic differences among the studied areas. All previous studies were conducted in the northern temperate regions of Iran, while the present one was conducted in the southern and tropical regions of the country.

The high level of BOD reported in the aquatic habitats of this species indicated the possibility of wastewater mixture with water resources in the region, ultimately leading to an increase in organic matter. It should be mentioned that the BOD and COD levels of the water in the *Cx. pipiens* larval habitats were checked and recorded for the first time in Iran. This species is one of the main vectors for transmission of WNV in different parts of the world. Considering Iran's conditions in terms of arboviral diseases, monitoring this vector seems necessary.

### 4.6 Culex antennatus

The study findings indicated that this species was only recorded at two high temperatures (78.3 % at 25 °C and 21.7 % at 26 °C). On the other hand, this water had the lowest pH. This result implied that an increase in temperature led to an increase in the abundance of mosquito larvae. On the contrary, a decrease in pH led to an increase in the frequency of larvae. Similar results were obtained in three different studies in Egypt, as well [[51], [52], [53]]. However, Hanafi-Bojd et al. (2017) did not report the physicochemical factors for *Cx. antennatus* due to the low abundance of this species in Hormozgan province. The current study measured and reported these factors in the oviposition site of this species for the first time in Iran.

### 4.7 Culex perexiguus

Similar to the results of physical analyses in the present investigation, Hanfi-Bojd et al. (2017) reported this species in permanent water (92.2 %), fully sun-lighted sites (53.8 %), partially sun-lighted sites (46.2 %), clear water (100 %), natural sites (50 %), and manmade sites (50 %) in southern Iran [20]. In 2015, Laddoni et al. indicated that 100 % of *Cx. perexiguus* was found in rice fields as a natural site in Isfahan province [50]. Omrani et al. (2020) also collected this species larvae from natural sites (58.3 %), artificial sites (41.7 %), temporary water (69.4 %), clear water (91.7 %), and sun-lighted sites (86.1 %) in Chaharmahal and Bakhtiari province [27]. In 2018, Sofizadeh et al. collected most of these larvae from the temporary and clear water in artificial sites in Golestan province [46]. According to these investigations, this species tends to lay eggs in clear water. Nevertheless, the results might have been affected by other physical parameters, such as sample size, geographical differences, and other effective variables.

Hanafi-Bojd et al. (2017) collected this species from a pH of 8.1 ± 0.4, TDS = 1262.9 ± 357.7 mg/L, EC = 2225.8 ± 232.7 μS/cm, NTU = 9.1 ± 2.1, alkalinity = 468.3 ± 63.4 mg/L, and total hardness = 291.5 ± 113.3 mg/L in Hormozgan province. They reported a high level of alkalinity (412.4–523 mg/L) and the lowest turbidity (NTU = 7.3) for the oviposition sites of this species in comparison to other species [20]. Considering the results of both studies, it can be noted that *Cx. perexiguus* females tend to lay eggs in clear aquatic habitats with a low level of turbidity.

Hanafi-Bojd et al. (2017) revealed a high level of total hardness and alkalinity in comparison to other species. In the current study, the largest number of larvae was reported from larval habitats with a low level of BOD. It should be noted that BOD and COD were measured for *Cx. perexiguus* larvae for the first time in Iran. Based on the results, 57.8 % of the larvae were collected from well water. Generally, *Cx. perexiguus* has an important role in the transmission of WNV [54]. Therefore, uncovered water wells can be a suitable environment for the growth and reproduction of this species. This point should be considered in the implementation of control strategies.

### 4.8 Diversity of the mosquito species

If a greater number of larvae had been collected from the selected sites, the dotted lines would have predicted the diversity of species. Overall, the results showed that the number of species was almost equal in the two communities, but the species tended to lay eggs in natural rather than manmade sites. Yet, this might have been influenced by many other factors.

### 4.9 The correlation between species

The study findings revealed a significant correlation between some collected species. In the same line, Hanfi-Bojd et al. (2017) recorded 36.6 % of the species only from Hormozgan province [20]. In 2013, Banafshi et al. also reported 96.6 % of this larvae from Kurdistan province [55]. Furthermore, Azari-Hamidian et al. (2005) collected *Cs. longiareolata* as the loneliest species in 10.6 % of cases [44]. These results showed that this species had a predatory behavior on other mosquito larvae.

In the current research, an increase in the number of *Cx. perexiguus* larvae led to an increase in the number of *Cx. antennatus* in Khoshk River and that of *Cx. quinquefasciatus* in School of Public Health. The significant affinity among these species showed that they probably did not have interspecific competition and were able to share their habitats with each other. This phenomenon occurs when there is enough food and space for all species. Further ecological studies are needed to examine these interspecific relationships in details.

## 5 Conclusions

The ecological results demonstrated that the richness and diversity of species were higher and more stable in natural sites than in manmade places. In addition, there was a significant correlation between the species and some physicochemical parameters of the habitats (p < 0.05).

Overall, the optimum BOD, COD, alkalinity, TDS, EC, pH, and temperature for mosquitoes in the studied areas were 140 mg/L, 360 mg/L, 160 mg/L, 420 mg/L, 840 μS/cm, 8.3, and 24 °C, respectively. The results revealed an inverse relationship between the species diversity and total hardness of water. Moreover, most mosquito species in this geographical condition tended to live in manmade, sunny oviposition sites with temporary and turbid water. Furthermore, environmental factors, especially physicochemical parameters of aquatic habitats, played a key role in the bioecological characteristics of mosquitoes, such as habitat selection, abundance, and vectorial capacity. These data can provide a better understanding of the biology and ecology of mosquitoes as the most important group of disease vectors to humans and animals. Hence, the results can be used to apply some safer and more environmentally friendly alternative methods instead of routine chemical control, such as environmental modification and environmental manipulation of vectors, as an essential part of the integrated vector management program.

## Ethics approval and consent to participate

Not applicable.

## Consent for publication

Not applicable.

## Data availability statement

Data will be made available on request.

## Funding

Not applicable.

## CRediT authorship contribution statement

**Parisa Soltan-Alinejad:** Conceptualization, Data curation, Methodology, Writing – original draft. **Shima Bahrami:** Formal analysis, Investigation, Writing – original draft. **Davood Keshavarzi:** Investigation, Methodology, Software, Visualization, Writing – original draft. **Marziae Shahriari-Namadi:** Investigation, Methodology, Writing – original draft. **Amin Hosseinpour:** Data curation, Investigation, Methodology, Visualization, Writing – original draft. **Aboozar Soltani:** Conceptualization, Formal analysis, Funding acquisition, Methodology, Supervision, Validation, Writing – original draft, Writing – review & editing.

## Declaration of competing interest

None declared.

## List of abbreviations

(BOD): Biochemical oxygen demand
(COD): Chemical oxygen demand
(pH): Potential of hydrogen
(NTU): Nephelometric turbidity unit
(EC): Electrical conductivity
(TDS): Total dissolved solids
(N, N-diethyl-*p*-phenylenediamine): DPD
(SPSS): Statistical package for the social sciences
(spp.): Species
(SD): Standard deviation
(M): Mean
(mg/L): Milligrams per liter
(μS/cm): Microsiemens per centimeter
